# Characteristics of Genomic Alterations in Pericardial Effusion of Advanced Non-small Cell Lung Cancer

**DOI:** 10.3389/fgene.2022.850290

**Published:** 2022-05-12

**Authors:** Jiaxue He, Xintong Hu, Liguo Chen, Qiaoliang Liu, Yanfang Jiang

**Affiliations:** ^1^ Key Laboratory of Organ Regeneration and Transplantation of the Ministry of Education, Genetic Diagnosis Center, The First Hospital of Jilin University, Changchun, China; ^2^ College of Communication Engineering, Jilin University, Changchun, China

**Keywords:** non-small cell lung cancer, pericardial effusion, next-generation sequencing, actionable alterations, plasma cfDNA

## Abstract

**Background:** The feasibility and value of pericardial effusion as a liquid biopsy sample for actionable alteration detection in patients with non-small cell lung cancer (NSCLC) has not been adequately investigated. Here, we aim to reveal genomic alterations between pericardial effusion and paired tumor tissue, plasma (plasma cfDNA), and pleural effusion supernatant (PE-cfDNA) based on second-generation sequencing technology.

**Material and methods:** A total of 26 advanced NSCLC patients were retrospectively studied. The following samples were collected and sequenced using two targeted next-generation sequencing panels: pericardial effusion (*n* = 26), matched tumor tissue (*n* = 6), plasma (*n* = 16), and pleural effusion supernatant (*n* = 5).

**Results:** A total of 10 actionable alterations were identified in pericardial effusion of the NSCLC patients, including *MET* amplification, *EGFR* L858R, *EGFR* T790M, *EGFR* exon 19 deletion, *EGFR* L861Q, *KRAS* G12C, *EML4-ALK* (exon 18: exon 20) fusion, *EML4-ALK* (exon 20: exon 20) fusion, *EML4-ALK* (exon 6: exon 20) fusion, and *ERBB2* exon 20 insertion. All these actionable alterations harbored multiple drug-sensitive targets as well as several drug-resistant targets, such as *EGFR* T790M. Compared to plasma cfDNA of 16 patients, paired pericardial effusion had higher number of actionable alterations (*p* = 0.08) as well as higher percentage of the population with actionable alterations (*p* = 0.16). Moreover, 8 out of 10 actionable alterations with single nucleotide variations (SNVs) or insertions/deletions (indels) had a higher variant allele frequency (VAF) in pericardial effusion than plasma cfDNA. In addition, we identified two actionable alterations in paired pericardial effusion, which were absence in PE-cfDNA. Clearly, 2 out of 3 actionable alterations with SNVs/indels in pericardial effusion had a higher VAF than those in PE-cfDNA. Our finding suggested the importance of pericardial effusion in the optimal selection of patients for targeted therapy.

**Conclusion:** Among liquid biopsy specimens from the advanced NSCLC patients, pericardial effusion may be a better candidate for genomic profiling than plasma cfDNA, while it could serve as a supplement to PE-cfDNA in detecting actionable alterations. Therefore, pericardial effusion might provide a new alternative for selection of patients for better treatment management.

## Introduction

Lung cancer is the second most common cancer as well as the leading cause of cancer death worldwide (Sung et al., 2021). Numerous studies have shown that compared with traditional chemotherapy, molecular-targeted therapies for driver alterations in patients with non-small cell lung cancer (NSCLC) have improved the survival of patients (Mitsudomi et al., 2010; Rosell et al., 2012; Sequist et al., 2013; Hyman et al., 2015; Kris et al., 2015; Zhong et al., 2018). Hence, it has become increasingly important to incorporate molecular genetic testing into standard clinical care (Tong et al., 2019).

While tissue specimens are typically considered optimal for molecular testing (Imazio et al., 2020), plasma has been widely used for genetic testing in detecting genetic alterations for guiding personalized therapy, especially the targeted therapy in lung cancer patients. However, genetic alteration detection using plasma liquid biopsy is always challenging due to the limited sensitivity (Diehl et al., 2008). In addition to plasma-based cell free DNA (cfDNA), tumor-derived cfDNA presented in other types of body fluids, such as pleural effusion (PE) and cerebrospinal fluid, are currently being evaluated in clinical settings (Tu et al., 2021; Wang et al., 2021). It has been shown that PE-derived cfDNA is a more reliable source of tumor DNAs for genetic alteration profiling (Husain et al., 2017; Lee et al., 2018; Tu et al., 2021). Liquid biopsy using cfDNA provides a novel approach for cancer genotyping (Engels et al., 2019; Yang et al., 2020). Neoplastic pericardial effusion is a serious and common manifestation of advanced malignancies. Pericardial effusion has been shown to contain fragments of cfDNA released locally from tumor cells in various serous cavities, which confer important diagnostic, prognostic, and therapeutic information. Thus, pericardial effusion has become an optimal substrate for liquid biopsy testing. To date, the concordance and difference in genetic alteration profiles among pericardial effusion, tumor tissue, plasma cfDNA, and pleural effusion supernatant (PE-cfDNA) have not been well characterized in NSCLC patients. In the meantime, the value of pericardial effusion in personalized therapy for NSCLC patients remains to be determined.

In this study, we performed comparative studies on genetic alteration profiles and actionable alterations among pericardial effusion supernatant (pericardial effusion-cfDNA), pericardial effusion cell sediment (pericardial effusion-sDNA), tumor tissue, plasma cfDNA, and PE-cfDNA in 26 advanced NSCLC patients. The actionable alterations were identified by interrogating the OncoKB database (https://www.oncokb.org/) (Chakravarty et al., 2017). The present study could provide a better liquid biopsy platform for genetic alteration detection in NSCLC patients.

## Materials and Methods

### Study Design, Patient Characteristics and Biospecimens

This study recruited a total of 26 advanced NSCLC patients from in-patient department of the First Hospital of Jilin University between January 2016 and December 2020, including 20 cases with lung adenocarcinomas and 6 cases with lung squamous carcinoma. The diagnosis was made according to the Guidelines for the Diagnosis and Treatment Standardization of Lung Cancer (2011 Edition). All the patients were staged based on the tumor node metastasis (TNM) staging system as presented in the 2012 National Comprehensive Cancer Network (NCCN) Clinical Practice Guidelines. They were pathologically classified using the 2015 edition of the World Health Organization’s Lung Cancer Histology Classification (Nicholson et al., 2016; Rami-Porta et al., 2016; Zakowski, 2016; El-Sherief et al., 2017). The median age of all patients was 56.5 years (range 42–83). Among the 26 patients, 14 (54%) were men, and 12 (46%) were women. The clinical stage was IV in all 26 patients. Ten patients (38%) had a smoking history (Supplementary Table S1).

**FIGURE 1:**
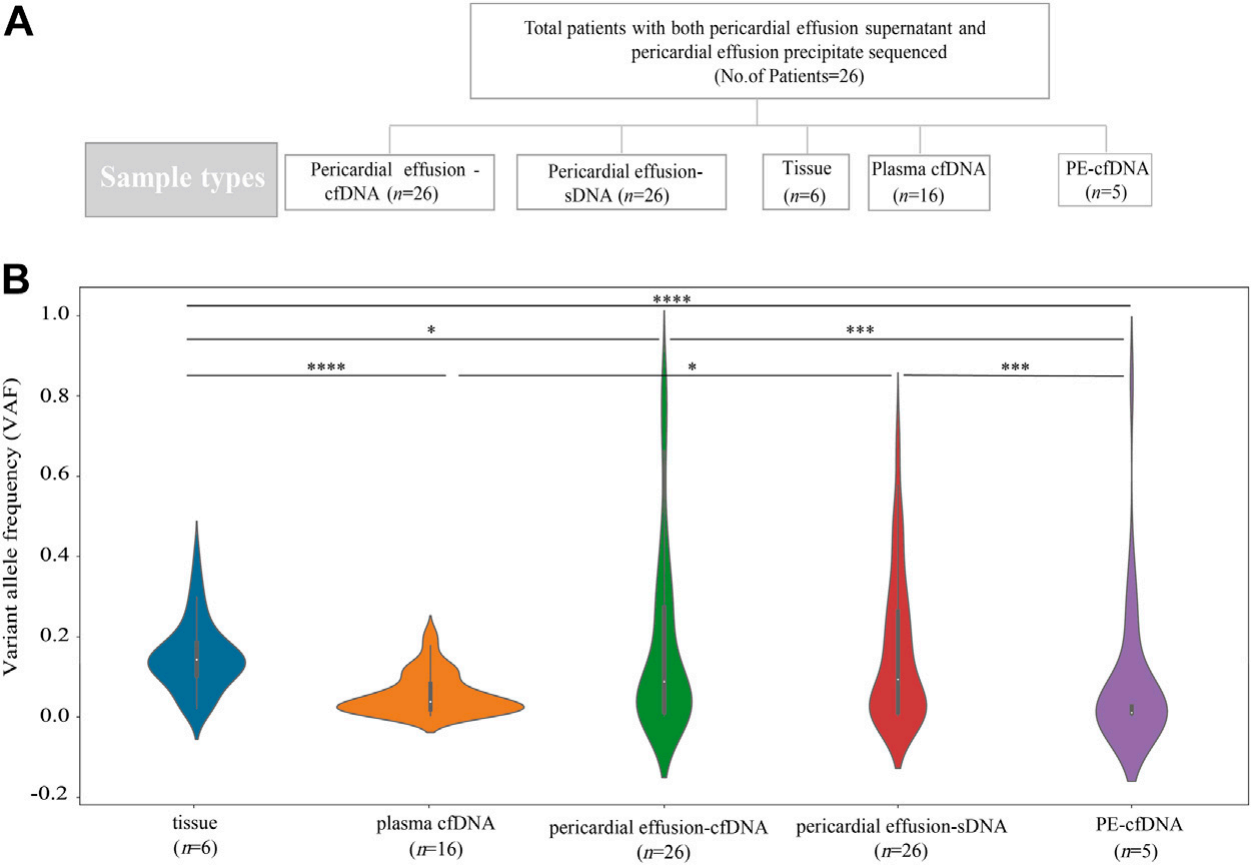
Sample information and VAFs of different sample types. **(A)** A total of 26 advanced NSCLC patients with pericardial effusion-cfDNA and pericardial effusion-sDNA were included in this study. Five biopsy specimens were sequenced and compared, including tumor tissue, plasma cfDNA, pericardial effusion-cfDNA, pericardial effusion-sDNA, and PE-cfDNA. **(B)** VAFs of tumor tissue, plasma cfDNA, pericardial effusion-cfDNA, pericardial effusion-sDNA, and PE-cfDNA.

We collected samples of matched pericardial effusion (*n* = 26), peripheral blood (*n* = 26), PE (*n* = 5), and tumor tissue (*n* = 6) from the NSCLC patients. 26 pericardial effusion specimens and 5 pleural effusion samples were subjected to centrifugation, and the cell pellets and supernatants were collected separately. In the meantime, 26 peripheral blood samples were centrifuged, and the plasmas and white blood cells were collected separately. NGS was performed to simultaneously detect gene alterations on pericardial effusion-cfDNA, pericardial effusion-sDNA, PE-cfDNA, plasma cfDNA, genomic DNA of white blood cells, and genomic DNA of formalin fixed paraffin-embedded (FFPE) tissue from NSCLC patients. Genomic DNA from the white blood cells of those 26 NSCLC patients was extraction as the germline controls for variant calling of other paired sample types. Plasma of 10 patients was excluded because of hemolysis in peripheral blood, insufficient plasma cfDNA extraction or failed quality control of sequencing data (Figure 1A; Supplementary Figure S1; Supplementary Table S2).

### Sample Preparation

Pericardial effusion, peripheral blood and PE were collected in 10 ml Cell-Free DNA Storage Tube (PET, cwbiotech) and centrifuged at 4000xg for 15 min at 4°C. Afterwards, 4 ml supernatant of pericardial effusion, 4 ml supernatant of PE and 4 ml plasma of peripheral blood were collected respectively for cfDNA extraction. The cell pellets of pericardial effusion were lysed with Proteinase K. The lysed cell pellets and white blood cells were used for genomic DNA extraction.

### DNA Extraction

CfDNA was extracted from supernatant of pericardial effusion, supernatant of PE and plasma of peripheral blood using MagMAX Cell-Free DNA isolation kit (Thermo Fisher Scientific, United States) and KingFisher™ Flex Magnetic Particle Processor 24DW (Thermo Fisher Scientific, United States) according to the user guide. Genomic DNA was extracted from the cell pellets of pericardial effusion and white blood cells with TIANamp Genomic DNA Kit (TIANGEN, Beijing, China). The blackPREP FFPE DNA Kit (Analytic jena, Germany) was used to extract genomic DNA from FFPE tumor tissue samples.

### Library Preparation

Genomic DNA from FFPE tumor tissue, white blood cells or cell pellets of pericardial effusion was sheared into 150 to 20 bp fragments using covaris M220 according to the recommended settings. CfDNA and fragmented DNA were input for library construction. Indexed Illumina NGS libraries were prepared using KAPA hyper preparation kit (Kapa Biosystems, United States) according to the manufacturer’s instructions. Ligated fragments were amplified for 9 PCR cycles using indexed primers depending on the DNA mass of pre-PCR. DNA was purified with Agencourt AMPure XP beads (Beckman-Coulter, United States), and dual size selection was performed during the library preparation. All the libraries were quantified with Qubit DNA dsDNA assay kit (Thermo Fisher, United States), and the fragment length was determined on Agilent bioanalyzer 2,100 using the DNA 1000 kit (Agilent, United States).

### Targeted Region Captures and Sequencing

Targeted region selection was performed using NimbleGen SeqCap Hybridization and Wash kit (Roche, Switzerland). 1 ug of mixed library DNA from 8-12 indexed Illumina libraries was captured with a hybridization probe. The probe library was designed through the NimbleDesign portal (Version 02) using genome build hg19 NCBI Build 37.1/GRCh37. DNA libraries were captured with two designed Genescope panels (Genecast, Beijing, China) including a total of 467 shared tumor-related genes. The captured products were quantified with Qubit dsDNA assay kit, subjected to determination of fragment length by Agilent 2,100 bioanalyzer with the DNA 1000 kit, and then sequenced using 150-bp paired-end runs on the Illumina Novaseq 6,000.

### Bioinformatics Pipeline

Data quality control, reference mapping and duplication masking were performed by Trimmomatic (version 0.36), BWA aligner (version 0.7.17) and Picard (version 2.23.0), respectively (Li et al., 2009; Li, 2013). Thereafter, realignment was carried out using Genome Analysis Tool Kit (version 3.7) (McKenna et al., 2010). Finally, processed BAM file was generated and used for subsequent analyses (Li et al., 2009; DePristo et al., 2011). We achieved a mean coverage depth of 6,561× across all target regions on all tissue samples, and a mean coverage depth of 3,950×, 3,429×, 4,324×, 7,321×, and 822× for pericardial effusion supernatant, pericardial effusion cell sediment, PE supernatant, plasma and matching white blood cells, respectively.

### Variant Calling

Single nucleotide variations (SNVs) and insertions/deletions (indels) were identified using VarDict (version 1.5.1) and FreeBayes (version 1.2.0) programs, and functional annotation of genetic variants was performed by ANNOVAR assay. To identify somatic SNVs and indels, matched white blood cell from each patients was used to filter the germline variants, sequencing artifacts and clonal hematopoiesis. Somatic genetic alterations including SNVs and indels were selected with the following filters: 1) those located in intergenic regions or intronic regions; 2) synonymous SNVs; 3) those with a minor allele frequency of >= 0.002 in database Exome Aggregation Consortum (ExAC) and genomad; 4) those with a variant allele frequency (VAF) of <0.003 in tumor tissue, plasma, pericardial effusion supernatant, pericardial effusion cell sediment, and PE supernatant; 5) strand bias for genetic alterations in the reads; 6) the number of supporting reads for a variation was <2; and 7) depth was <30x (Sherry et al., 2001; Wang et al., 2010; Forbes et al., 2011; Liu et al., 2011; Abecasis et al., 2012; Koboldt et al., 2012; Karczewski et al., 2017). The cnvkit software (version 0.9.2) was used to perform the copy number variation (CNV) calling from the tumor tissue, plasma, pericardial effusion supernatant, pericardial effusion cell sediment, and PE supernatant against the paired white blood cells. The copy number threshold for CNV gain and CNV loss was set at 2.5 and 1, respectively (Talevich et al., 2016). Somatic fusion genes were detected and filtered using Fusionmap with default parameters (Ge et al., 2011). The data of pericardial effusion were obtained from the merging of pericardial effusion-cfDNA and pericardial effusion-sDNA data. If the same alteration is shared in both pericardial effusion-cfDNA and pericardial effusion-sDNA, then the maximum VAF or copy number is taken as the VAF or copy number of this alteration in the pericardial effusion.

### Statistical Analysis

The coincidence rate of altered genes between the two groups of samples was defined as (number of patients with the shared altered gene in two groups of samples)/(total number of patients with the altered gene) * 100%. Statistical analyses were performed using R (version 3.6.3) or scipy. stats (version 1.3.1), statsmodels (version 0.10.1) and scikit_posthocs (version 0.6.7) packages in python (version 3.7.4). Fisher’s exact test was performed to evaluate differences between the proportions. Differences between multiple groups were compared using the Kruskal–Wallis test, and a comparison of two matched groups was made by post-hoc analyses using the Dunn’s test. Paired wilcoxon test was used to compare difference between paired samples of two groups. All the tests were two-sided, and *p* values <0.05 were regarded as statistically significant unless otherwise specified.

## Results

### Comparison of VAF for Overall Genetic Alterations Among the Different Sample Types

We compared the genetic alteration profiles among tumor tissue, plasma cfDNA, pericardial effusion-cfDNA, pericardial effusion-sDNA, and PE-cfDNA from 26 advanced NSCLC patients. As shown in Figure 1B, VAF of overall genetic alterations in pericardial effusion-sDNA (median 9.36%) was higher than that in plasma cfDNA (median 3.81%) or PE-cfDNA (median 1.06%), while it was much lower than that in tumor tissue (median 14.31%). Moreover, we observed that while VAF of overall genetic alterations in pericardial effusion-cfDNA (median 8.82%) was significantly higher than that in PE-cfDNA (median 1.06%). There was no significant difference in VAF of overall genetic alterations between pericardial effusion-cfDNA (median 8.82%) and pericardial effusion-sDNA (median 9.36%). These data suggested that pericardial effusion-sDNA may be a better specimen for detecting genetic alterations as compared to plasma cfDNA and PE-cfDNA.

### The Concordance and Difference in Genetic Alteration Profiling Between Pericardial Effusion-cfDNA and Pericardial Effusion-sDNA

**FIGURE 2:**
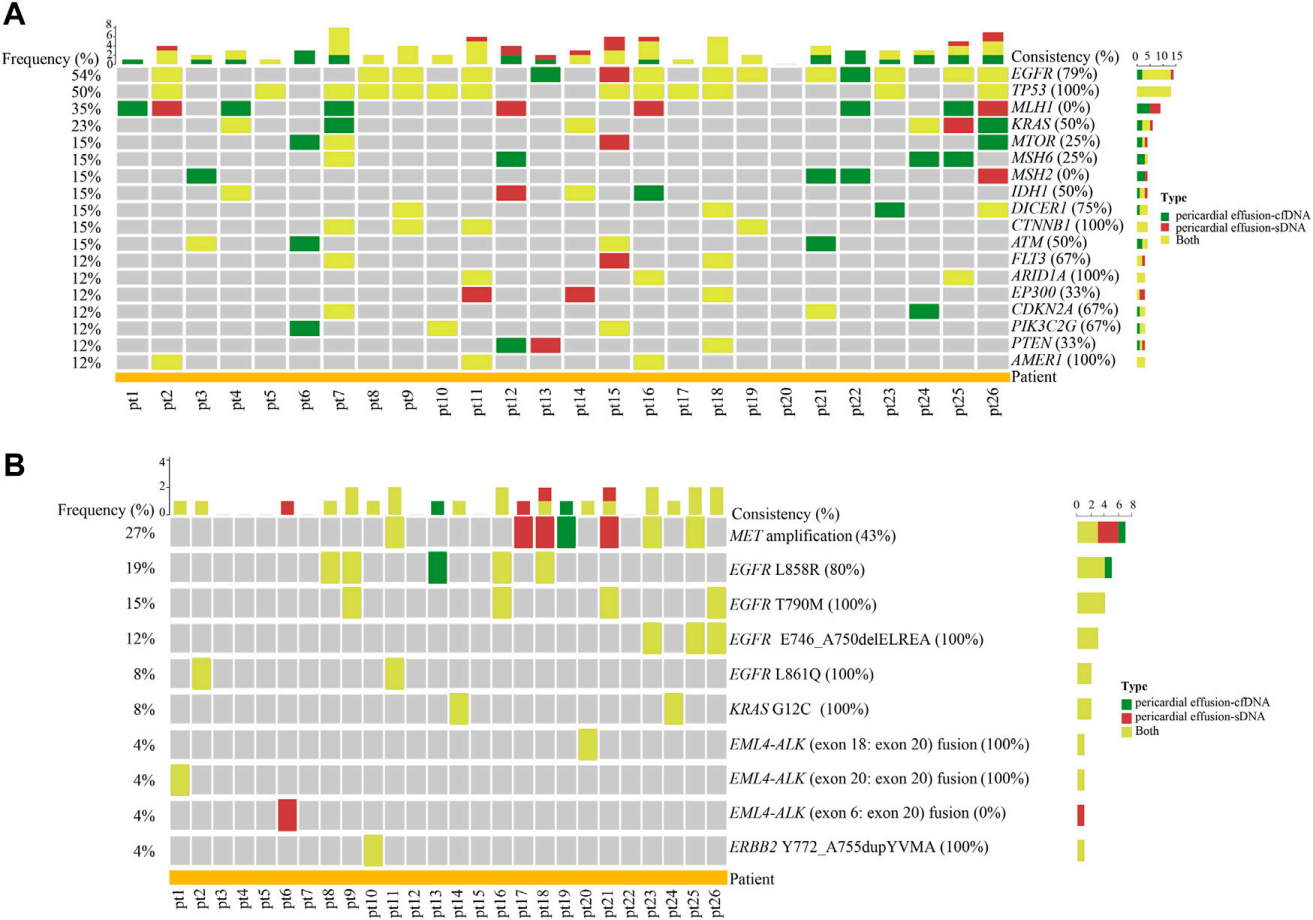
The concordance of gene alterations between pericardial effusion-cfDNA and pericardial effusion-sDNA. **(A)** Heatmap of the concordance of altered genes between pericardial effusion-cfDNA and the corresponding sDNA (only the genes with a total population frequency of more than 10% are displayed). **(B)** Landscape of actionable alterations in pericardial effusion-cfDNA and the corresponding sDNA. Altered genes and actionable alterations detected in pericardial effusion-cfDNA, pericardial effusion-sDNA, or pericardial effusion-cfDNA/pericardial effusion-sDNA were indicated by different colors.

We further analyzed the consistence and difference of altered genes between pericardial effusion-sDNA and pericardial effusion-cfDNA. As illustrated in Figure 2A, we identified 18 altered genes with the frequency of more than 10% in pericardial effusion-cfDNA and pericardial effusion-sDNA, which included *EGFR* (54%), *TP53* (50%), *MLH1* (35%), *KRAS* (23%), *MTOR* (15%), *MSH6* (15%), *MSH2* (15%), *IDH1* (15%), *DICER1* (15%), *CTNNB1* (15%), *ATM* (15%), *FLT3* (12%), *ARID1A* (12%), *EP300* (12%), *CDKN2A* (12%), *PIK3C2G* (12%), *PTEN* (12%), and *AMER1* (12%). Among them, 4 genes including *TP53*, *CTNNB1*, *ARID1A*, and *AMER1* were altered simultaneously in paired pericardial effusion-cfDNA and pericardial effusion-sDNA. In addition, the coincidence rate of altered genes *EGFR*, *DICER1*, *FLT3*, *CDKN2A*, *PIK3C2G*, *KRAS*, *IDH1* and *ATM* between pericardial effusion-cfDNA and pericardial effusion-sDNA ranged from 50% (50% included) to 100%. Notably, genetic alterations of *MLH1* and *MSH2* were detected exclusively in pericardial effusion-cfDNA or pericardial effusion-sDNA.

As depicted in Figure 2B, a total of 10 actionable alterations were identified in pericardial effusion-cfDNA and pericardial effusion-sDNA of the 26 patients, including *MET* amplification (27%), *EGFR* L858R (19%), *EGFR* T790M (15%), *EGFR* E746_A750delELREA (exon 19 deletion, 12%), *EGFR* L861Q (8%), *KRAS* G12C (8%), *EML4-ALK* (exon 18: exon 20) fusion (4%), *EML4-ALK* (exon 20: exon 20) fusion (4%), *EML4-ALK* (exon 6: exon 20) fusion (4%), and *ERBB2* Y772_A755dupYVMA (4%). Among them, *EGFR* T790M, *EGFR* E746_A750delELREA, *EGFR* L861Q, *KRAS* G12C, *EML4-ALK* (exon 18: exon 20) fusion, *EML4-ALK* (exon 20: exon 20) fusion, and *ERBB2* Y772_A755dupYVMA were detected in both pericardial effusion-cfDNA and pericardial effusion-sDNA. Meanwhile, *EGFR* L858R and *MET* amplification remained a modest consistence (40–80%) between paired pericardial effusion-cfDNA and pericardial effusion-sDNA. On the contrary, *EML4-ALK* (exon 6: exon 20) were found only in pericardial effusion-sDNA. All these data indicated that pericardial effusion-cfDNA and pericardial effusion-sDNA are complementary for detecting altered genes and actionable alterations in the advanced NSCLC patients.

Given the difference and overlap of detected genetic alterations between pericardial effusion-cfDNA and pericardial effusion-sDNA, we combined the variations of pericardial effusion-cfDNA and the corresponding sDNA in subsequent comparisons with other sample types.

### The Concordance and Difference in Genetic Alteration Profiling Between Tumor Tissue and Pericardial Effusion

**FIGURE 3:**
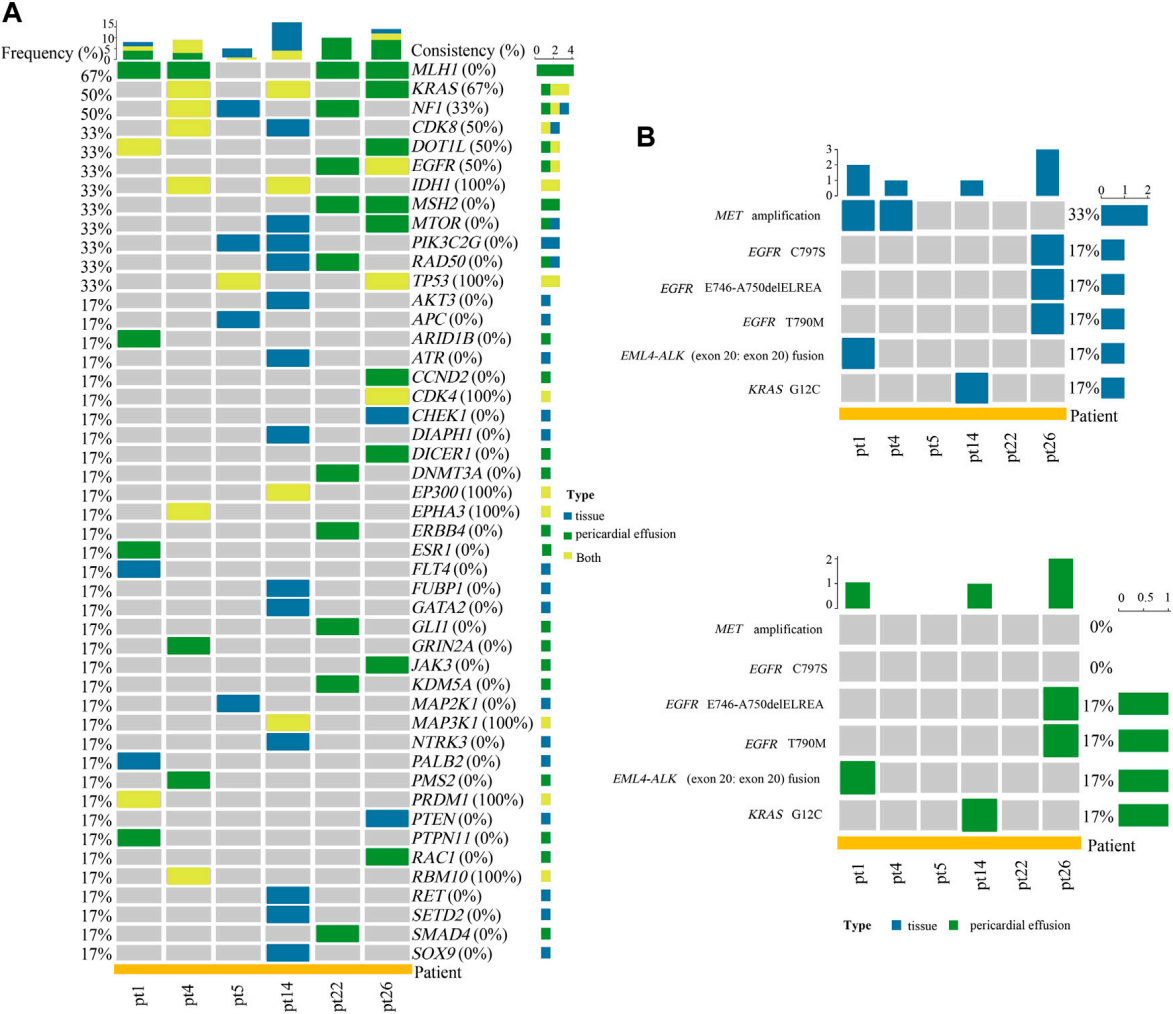
Analyses of gene alterations in tumor tissue and pericardial effusion. **(A)** Heatmap of the concordance of altered genes between tumor tissue and pericardial effusion. **(B)** Landscape of actionable alterations in tumor tissue and pericardial effusion. Altered genes and actionable alterations detected in tumor tissue, pericardial effusion, or tumor tissue/pericardial effusion were indicated by different colors.

Among the 26 advanced NSCLC patients with pericardial effusion, 6 had paired primary tumor tissues. As shown in Figure 3A, a total of 47 altered genes were identified in the 6 pairs of tumor tissue and pericardial effusion. Among them, *IDH1*, *TP53*, *CDK4*, *EP300*, *EPHA3*, *MAP3K1*, *PRDM1*, and *RBM10* displayed a 100% coincidence rate of genetic alteration between pericardial effusion and paired tumor tissue, while *KRAS*, *CDK8*, *DOT1L*, and *EGFR* had a coincidence rate of 50% or more. In addition, *NF1* was found to have a coincidence rate of 33%. Notably, 16 genes were differentially altered in the tumor tissue and pericardial effusion. As illustrated in Figure 3B, a total of 6 actionable alterations were identified, including *MET* amplification (33 vs. 0%), *EGFR* C797S (17 vs. 0%), *EGFR* E746_A750delELREA (17 vs. 17%), *EGFR* T790M (17 vs. 17%), *EML4-ALK* (exon 20: exon 20) fusion (17 vs. 17%), and *KRAS* G12C (17 vs. 17%). Among the actionable alterations, *MET* amplification exhibited the highest population frequency, while *EGFR* E746_A750delELREA, *EGFR* T790M, *EML4-ALK* (exon 20: exon 20) fusion, and *KRAS* G12C displayed a 100% consistency between the tumor tissue and pericardial effusions. Besides, *MET* amplification and *EGFR* C797S were detected only in the tumor tissues. The patient (pt26) carrying the *EGFR* C797S alteration had both *EGFR* 19del and *EGFR* T790M alterations detected, consistent with the patient’s history of treatment with icotinib and osimertinib.

### Pericardial Effusion May Harbor More Tissue-Derived Gene Variations Than Plasma cfDNA

**FIGURE 4:**
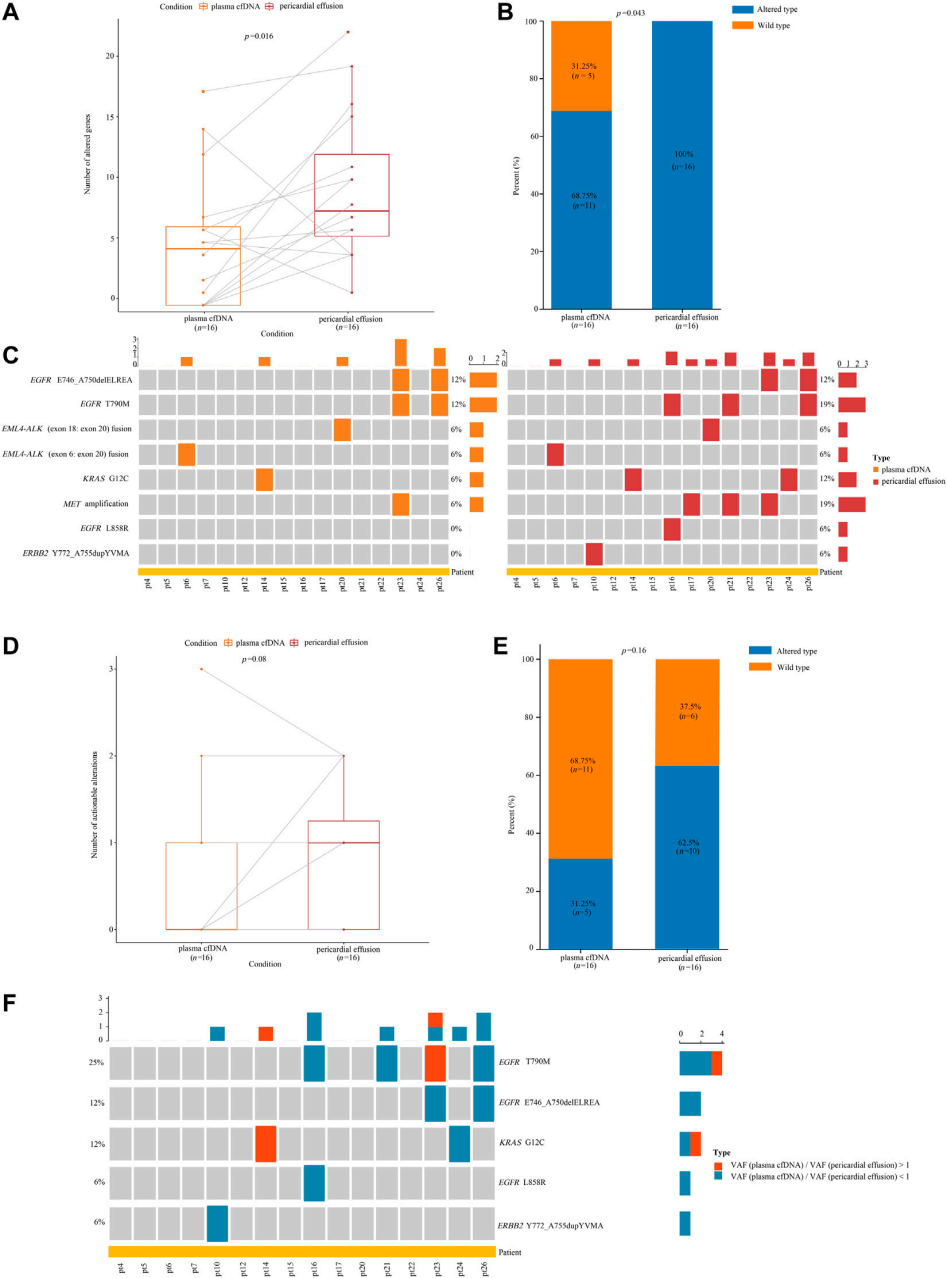
Analyses of gene alterations in pericardial effusion and plasma cfDNA. **(A)** Comparison of the number of altered gene between pericardial effusion and plasma cfDNA. **(B)** The frequencies of altered genes in pericardial effusion and plasma cfDNA. **(C)** Landscape of actionable alterations in pericardial effusion and plasma cfDNA. **(D)** Comparison of the numbers of actionable alterations between pericardial effusion and plasma cfDNA. **(E)** The frequencies of actionable alterations in pericardial effusion and plasma cfDNA. **(F)** Comparison of the VAF of actionable alterations with SNVs/indels between pericardial effusion and plasma cfDNA.

Among the 26 advanced NSCLC patients with pericardial effusion, 16 had paired plasma samples. We first compared the genetic alteration profiles between pericardial effusion and plasma cfDNA. As depicted in Figures 4A,B, the number (*p* = 0.016) of altered genes and the genetic alteration rate (*p* = 0.043) were significantly increased in pericardial effusion compared with plasma cfDNA. We next examined actionable alterations from paired plasma cfDNA and pericardial effusion. As illustrated in Figure 4C, a total of 8 actionable alterations were identified in plasma cfDNA and pericardial effusion, including *EGFR* E746_A750delELREA (12 vs. 12%), *EGFR* T790M (12 vs. 19%), *EML4-ALK* (exon 18: exon 20) fusion (6 vs. 6%), *EML4-ALK* (exon 6: exon 20) fusion (6 vs. 6%), *KRAS* G12C (6 vs. 12%), *MET* amplification (6 vs. 19%), *EGFR* L858R (0 vs. 6%), and *ERBB2* Y772_A755dupYVMA (0 vs. 6%). Clearly, pericardial effusion harbored a higher number of actionable alterations than the corresponding plasma cfDNA with a certain trend toward significance (*p* = 0.08) (Figure 4D). Moreover, the rate of actionable alterations in pericardial effusion was higher than that in plasma cfDNA, albeit there was a near-marginal significance (*p* = 0.16) (Figure 4E). Besides, we identified a total of 10 actionable alterations involving SNVs or indels in the samples. Among them, 8 (80%) were found to have a higher VAF in pericardial effusion compared with plasma cfDNA (Figure 4F). Together, these results suggested that pericardial effusion may harbor more tissue-derived gene variations.

### The Concordance and Difference in Actionable Alterations Between Pericardial Effusion and PE-cfDNA

**FIGURE 5:**
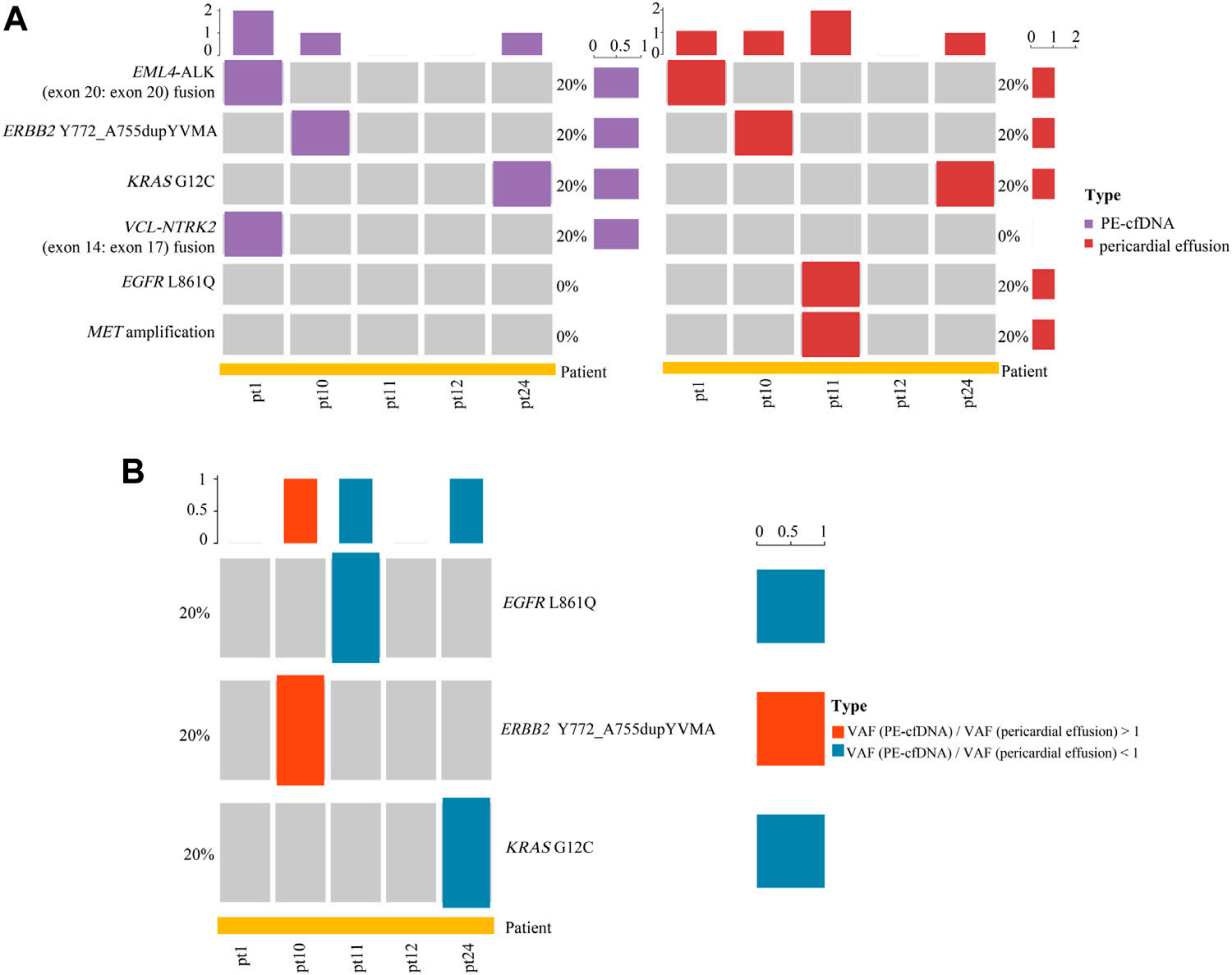
Analyses of actionable alterations in PE-cfDNA and pericardial effusion. **(A)** Landscape of actionable alterations in PE-cfDNA and pericardial effusion. **(B)** Comparison of the VAF of actionable alterations with SNVs/indels between PE-cfDNA and pericardial effusion.

It has been reported that PE-cfDNA (pleural effusion supernatant) is a better source of tissue-derived gene alterations than plasma cfDNA (Tu et al., 2021). Paired samples of pericardial effusion and PE-cfDNA from 5 advanced NSCLC patients were analyzed for actionable alterations. As shown in Figure 5A, a total of 6 actionable alterations were identified in PE-cfDNA and pericardial effusion, including *EML4-ALK* (exon 20: exon 20) fusion (20 vs. 20%), *ERBB2* Y772_A755dupYVMA (20 vs. 20%), *KRAS* G12C (20 vs. 20%), *VCL-NTRK2* (exon 14: exon 17) fusion (20 vs. 0%), *EGFR* L861Q (0 vs. 20%), and *MET* amplification (0 vs. 20%). Among the 6 actionable alterations, *EML4-ALK* (exon 20: exon 20) fusion, *ERBB2* Y772_A755dupYVMA and *KRAS* G12C showed a consistency of 100% between PE-cfDNA and pericardial effusion. Moreover, we identified *EGFR* L861Q and *MET* amplification as two unique actionable alterations in pericardial effusion, suggesting that NGS sequencing on pericardial effusion could lead to identification of advanced NSCLC patients who are qualified for TKI based therapy. Notably, 3 out of the 6 actionable alterations were found to harbor SNVs/indels, including *EGFR* L861Q, *ERBB2* Y772_A755dupYVMA and *KRAS* G12C. Among the 3 actionable alterations with SNVs/indels, 2 (66.7%) (*EGFR* L861Q and *KRAS* G12C) had a higher VAF in pericardial effusion compared with PE-cfDNA (Figure 5B).

## Discussion

As a non-invasive method, liquid biopsy has been used in clinical practice for monitoring the tumor treatment efficacy and occurrence of drug resistance (Rossi and Ignatiadis, 2019; Siravegna et al., 2019). Plasma-based genotyping has demonstrated its value in guiding personalized treatment of lung cancer patients (Aggarwal et al., 2019; Sabari et al., 2019). While pericardial effusion is considered a clinically accessible body fluid, its value in guiding personalized therapy has yet to be determined. Herein, we performed targeted sequencing to analyze 4 different types of samples from patients with stage IV NSCLC. The present study showed that while pericardial effusion-ctDNA had a higher VAF of overall genetic alterations than plasma cfDNA and PE-cfDNA, pericardial effusion was in a good agreement with the tumor tissue in detection of actionable alterations. Moreover, we found that pericardial effusion-cfDNA and pericardial effusion-sDNA from the NSCLC patients are complementary for detecting actionable alterations. Further studies suggested that pericardial effusion might be a better source of tissue-derived gene alterations than plasma cfDNA. In addition, the comparative analysis of PE and pericardial effusion samples of 5 patients suggested that for patients whose pericardial effusion samples are accessible, pericardial effusion samples should be tested at the same time.

Pericardial effusion is among liquid biopsy specimens for detection of genetic variants. In this study, we compared the genetic alteration profiles between tumor tissue and pericardial effusion from the NSCLC patients and found that both shared and unique altered genes were detected in each specimen. This finding indicates that pericardial effusion harbors unique altered genes that were absent in matched tumor tissues, suggesting that pericardial effusion is representative of the tumor heterogeneity in NSCLC. Moreover, we observed a 57.1% (4/7) coincidence rate of actionable alterations between pericardial effusion and tumor tissue, indicating that pericardial effusion could be an alternative source of tumor-derived DNAs for genetic alteration profiling and for guiding targeted therapy as well as a fluid biopsy sample.

The present study showed that compared with plasma, pericardial effusion had a greater capability for detecting altered genes and actionable alterations. Although plasma can be used to guide targeted therapy in NSCLC patients whose tumor tissue is not accessible for biopsy, plasma cfDNA accounts for only approximately 0.01% of tumor cfDNA (Lee et al., 2016). In this study, we found that pericardial effusion had a higher VAF of overall genetic alterations and actionable alterations as well as a higher number and rate of altered genes and actionable alterations than plasma cfDNA. The above finding demonstrates that pericardial effusion is more effective and accurate in detecting genetic variants than plasma cfDNA. Hence, pericardial effusion could potentially serve as an alternative of plasma in clinical practice. For NSCLC patients whose tumor tissue is not easily accessible for biopsy, pericardial effusion may be superior to plasma in guiding personalized therapy.

Multiple studies have shown that PE-cfDNA is superior to plasma as a liquid biopsy specimen. In these cases, a more comprehensive genetic alteration profile was detected in PE-cfDNA as compared to plasma samples (Tong et al., 2019). Moreover, PE-cfDNA displayed a significantly higher median mutant allele frequency and higher coincidence rate of overall genetic alterations with tumor tissue than plasma cfDNA (Tu et al., 2021). In the current study, we showed that the VAF of overall genetic alterations in pericardial effusion-cfDNA or pericardial effusion-sDNA was significantly higher than that in PE-cfDNA, while 2 out of 3 actionable alterations with SNVs/indels had a higher VAF in pericardial effusion compared with PE-cfDNA. A high consistency of actionable alterations between pericardial effusion and PE-cfDNA as well as identification of unique actionable alterations for each specimen suggested that pericardial effusion may be a good complement to PE-cfDNA in detecting actionable alterations in patients with no accessible tumor tissue for biopsy.

There were still several limitations in this study. First, the sample size needs to be increased to obtain results with a high confidence. Second, lack of detailed treatment history and follow up of patients prevented us from evaluating and comparing therapeutic outcomes among the different treatment options based on pericardial effusion, plasma cfDNA or PE-cfDNA.

## Conclusion

Among liquid biopsy specimens from the advanced NSCLC patients, pericardial effusion could be a better candidate for genomic profiling than plasma cfDNA, while it may serve as a complement to PE-cfDNA in detecting actionable alterations.

## Data Availability

The datasets presented in this study can be found in online repositories. The names of the repository/repositories and accession number(s) can be found below: https://ngdc.cncb.ac.cn/gsa/ HRA002198.

## Supplementary Material

Supplementary Figure S1 Flow chart of the study.
