# Supplementary material for: Characteristics of Genomic Alterations in Pericardial Effusion of Advanced Non-small Cell Lung Cancer
Source: Front Genet. 2022 May 12;13:850290. doi: 10.3389/fgene.2022.850290 (PMC9133843; doi:10.3389/fgene.2022.850290)
Supplement: Supplementary file 1 [file Table1.docx]

**Table S1. Clinical characteristics of patients**

| **Characteristic** | **All patients (n=26)** |  |
| --- | --- | --- |
| **Age (years)** |  |  |
| Median (range) | 56.5 (42-83) |  |
| **Sex (n, %)** |  | |
| Male | 14 (54%) |  |
| Female | 12 (46%) |  |
| **Histological types (n, %)** |  |  |
| Adenocarcinoma | 20 (77%) |  |
| Squamous cell carcinoma | 6 (23%) |  |
| **Clinical stage (n, %)** |  |  |
| IV | 26 (100%) |  |
| **Smoking history (n, %)** |  |  |
| Yes | 10 (38%) |  |
| No | 16 (62%) |  |
