# Supplementary figures and images for: Characteristics of Genomic Alterations in Pericardial Effusion of Advanced Non-small Cell Lung Cancer
Source: Front Genet. 2022 May 12;13:850290. doi: 10.3389/fgene.2022.850290 (PMC9133843; doi:10.3389/fgene.2022.850290)

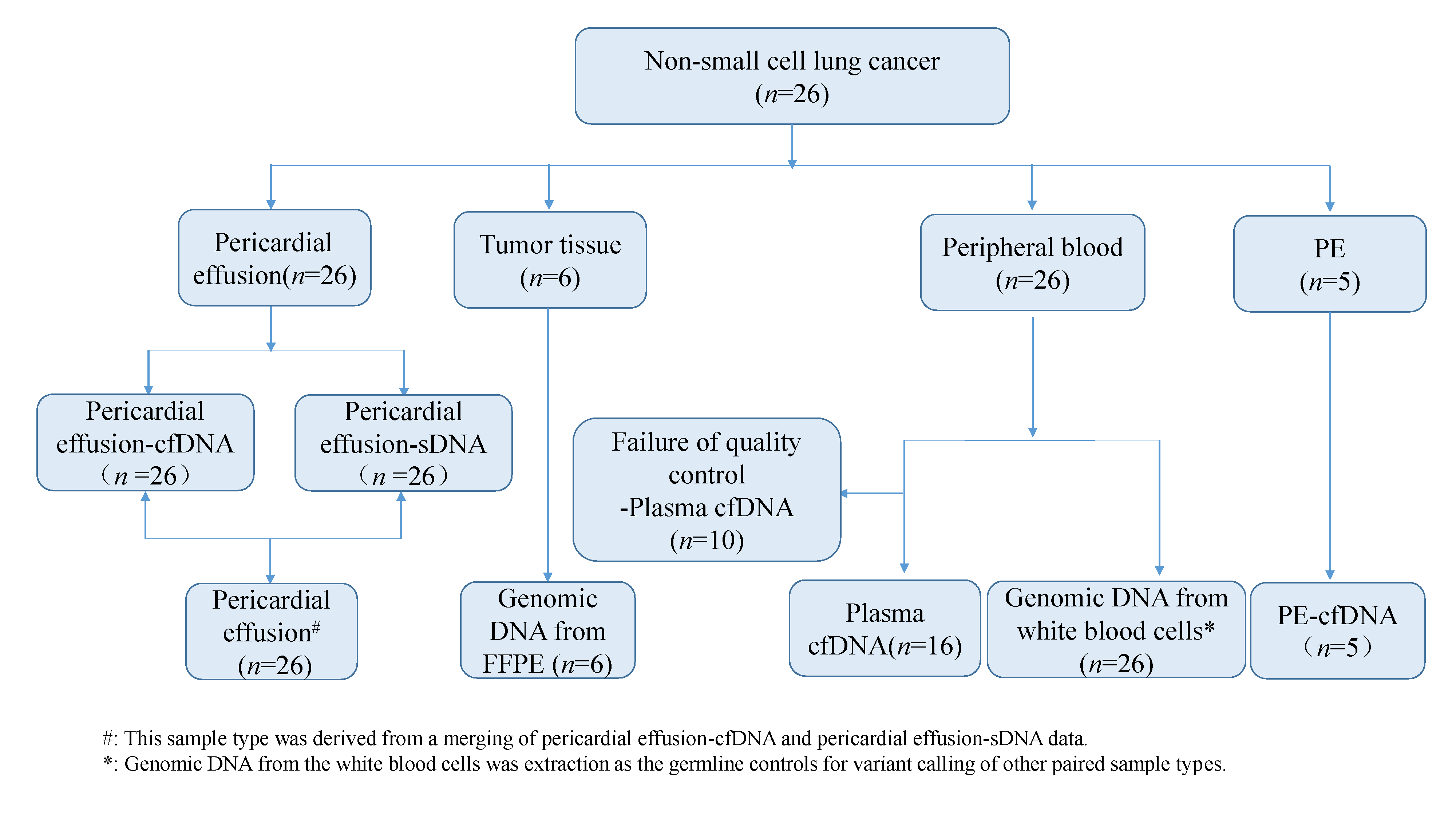

Supplement: Supplementary file 2 [file Image1.TIF]
