# Supplementary material for: Characteristics of Genomic Alterations in Pericardial Effusion of Advanced Non-small Cell Lung Cancer
Source: Front Genet. 2022 May 12;13:850290. doi: 10.3389/fgene.2022.850290 (PMC9133843; doi:10.3389/fgene.2022.850290)
Supplement: Supplementary file 3 [file Table2.DOCX]

TableS2. Details of sample type and number

| **Patient (n)** | **pericardial**  **effusion-cfDNA^a^** | **pericardial**  **effusion-sDNA^a^** | **pericardial**  **effusion^b^** |  | **White blood**  **cells^c^** | **Tumor**  **tissue** | **Plasma**  **cfDNA** | **PE**  **-cfDNA** |
| --- | --- | --- | --- | --- | --- | --- | --- | --- |
| pt1 | √ | √ | √ |  | √ | √ |  | √ |
| pt2 | √ | √ | √ |  | √ |  |  |  |
| pt3 | √ | √ | √ |  | √ |  |  |  |
| pt4 | √ | √ | √ |  | √ | √ | √ |  |
| pt5 | √ | √ | √ |  | √ | √ | √ |  |
| pt6 | √ | √ | √ |  | √ |  | √ |  |
| pt7 | √ | √ | √ |  | √ |  | √ |  |
| pt8 | √ | √ | √ |  | √ |  |  |  |
| pt9 | √ | √ | √ |  | √ |  |  |  |
| pt10 | √ | √ | √ |  | √ |  | √ | √ |
| pt11 | √ | √ | √ |  | √ |  |  | √ |
| pt12 | √ | √ | √ |  | √ |  | √ | √ |
| pt13 | √ | √ | √ |  | √ |  |  |  |
| pt14 | √ | √ | √ |  | √ | √ | √ |  |
| pt15 | √ | √ | √ |  | √ |  | √ |  |
| pt16 | √ | √ | √ |  | √ |  | √ |  |
| pt17 | √ | √ | √ |  | √ |  | √ |  |
| pt18 | √ | √ | √ |  | √ |  |  |  |
| pt19 | √ | √ | √ |  | √ |  |  |  |
| pt20 | √ | √ | √ |  | √ |  | √ |  |
| pt21 | √ | √ | √ |  | √ |  | √ |  |
| pt22 | √ | √ | √ |  | √ | √ | √ |  |
| pt23 | √ | √ | √ |  | √ |  | √ |  |
| pt24 | √ | √ | √ |  | √ |  | √ | √ |
| pt25 | √ | √ | √ |  | √ |  |  |  |
| pt26 | √ | √ | √ |  | √ | √ | √ |  |

a: The data of this sample type is analyzed separately.

b: This sample type was derived from a merging of pericardial effusion-cfDNA and pericardial effusion-sDNA data.

c: Genomic DNA from the white blood cells was extraction as the germline controls for variant calling of other paired sample types.

√: The patient has one sample of this sample type.
